# Structural and Stress Properties of AlGaN Epilayers Grown on AlN-Nanopatterned Sapphire Templates by Hydride Vapor Phase Epitaxy

**DOI:** 10.3390/nano8090704

**Published:** 2018-09-10

**Authors:** Chi-Tsung Tasi, Wei-Kai Wang, Sin-Liang Ou, Shih-Yung Huang, Ray-Hua Horng, Dong-Sing Wuu

**Affiliations:** 1 Department of Materials Science and Engineering, National Chung Hsing University, Taichung 40227, Taiwan; d100066018@mail.nchu.edu.tw; 2 Department of Materials Science and Engineering, Da-Yeh University, Changhua 51591, Taiwan; wk@mail.dyu.edu.tw (W.-K.W.); slo@mail.dyu.edu.tw (S.-L.O.); 3 Department of Industrial Engineering and Management, Da-Yeh University, Changhua 51591, Taiwan; syh@mail.dyu.edu.tw; 4 Department of Electronics Engineering, National Chiao Tung University, Hsinchu 300, Taiwan; rhh@nctu.edu.tw; 5 Research Center for Sustainable Energy and Nanotechnology, National Chung Hsing University, Taichung 40227, Taiwan; 6 Innovation and Development Center of Sustainable Agriculture, National Chung Hsing University, Taichung 40227, Taiwan

**Keywords:** AlGaN, nanopatterned sapphire substrate, hydride vapor phase epitaxy, stress, transmission electron microscopy

## Abstract

In this paper, we report the epitaxial growth and material characteristics of AlGaN (Al mole fraction of 10%) on an AlN/nanopatterned sapphire substrate (NPSS) template by hydride vapor phase epitaxy (HVPE). The crystalline quality, surface morphology, microstructure, and stress state of the AlGaN/AlN/NPSS epilayers were investigated using X-ray diffraction (XRD), atomic force microscopy (AFM), and transmission electron microscopy (TEM). The results indicate that the crystal quality of the AlGaN film could be improved when grown on the AlN/NPSS template. The screw threading dislocation (TD) density was reduced to 1.4 × 10^9^ cm^−2^ for the AlGaN epilayer grown on the AlN/NPSS template, which was lower than that of the sample grown on a flat c-plane sapphire substrate (6.3 × 10^9^ cm^−2^). As examined by XRD measurements, the biaxial tensile stress of the AlGaN film was significantly reduced from 1,187 MPa (on AlN/NPSS) to 38.41 MPa (on flat c-plane sapphire). In particular, an increase of the Al content in the overgrown AlGaN layer was confirmed by the TEM observation. This could be due to the relaxation of the in-plane stress through the AlGaN and AlN/NPSS template interface.

## 1. Introduction

AlGaN ternary alloy templates have recently drawn increasing attention because of their potential in expanding the fabrication of optoelectronic devices operating in the ultraviolet (UV) range and high-power, high-frequency electronic devices [1,2,3,4,5]. Because of a critical lattice mismatch between the Al*_x_*GaN_1−*x*_ and the sapphire, heteroepitaxial growth-induced defects, such as threading dislocations (TDs), voids, and stacking faults, are usually observed [6,7] on the upper grown layer, hence destroying the performance of UV devices drastically [8,9,10,11]. Therefore, the epitaxial growth of thick, crack-free, high-quality AlGaN with a low dislocation density template plays an important role in constructing high-performance AlGaN-based optoelectronic devices. The hydride vapor phase epitaxy (HVPE) method has been shown to achieve the growth of a thick AlGaN layer serving as a template (or bulk) substrate material due to its rapid growth rate (several hundred μm/h) and relatively low cost [12,13]. However, due to the significant lattice mismatch between the AlGaN and the sapphire, the crystalline quality of the HVPE AlGaN with a low defect density is unsatisfactory. Meanwhile, epilayer cracks are induced when the critical thickness of AlGaN is exceeded during the cooling down procedure. Epitaxial lateral overgrowth (ELOG) techniques on microstripe (or honeycomb) shape-patterned sapphires have shown a promising result in reducing the defect density of the AlGaN layer [14,15,16]. In addition, the uses of nanopatterned sapphire substrates (NPSSs) improve the crystalline quality of the AlGaN layer by ELOG [17]. Published research using in situ AlN buffer layer below the grown Al_0.45_Ga_0.55_N layer showed that it could not only enhance the crystallinity but also affect the surface morphology due to the misorientated crystallites [18]. The effect of various growth temperatures and V/III ratios of the AlN buffer layer on the structural properties of the subsequently grown AlGaN layer has been reported [19,20]. Another major issue is the low efficiency of Al incorporation in Al*_x_*Ga_1−*x*_N caused by biaxial tensile strain formation during the growing process [21]. This limited the efforts on the study of high Al content of AlGaN films and crystalline quality. It has been previously reported that high temperature growth of AlN film is considered to serve as a strain-relaxed layer to improve nitride material’s structural properties [22,23]. Therefore, it is important to grow high Al content Al*_x_*Ga_1−*x*_N films with low defect density by the above-mentioned method. Several groups have demonstrated the AlN template/NPSS by subsequently growing UV devices by metalorganic chemical vapor deposition (MOCVD) [24,25,26]. Since the considerable production cost of MOCVD growth AlGaN template would be too much, HVPE method to fabricate AlGaN templates on foreign substrates are good choices for the heteroepitaxial deposition of AlGaN-based devices. In this study, the AlGaN layer was grown in a combination of ex situ MOCVD grown AlN buffer layer and NPSS surface by HVPE. In addition, the growth mechanism, crystalline quality, surface morphology, and structural properties of the AlGaN on the AlN/NPSS template were investigated.

## 2. Materials and Methods

**Figure 1:**
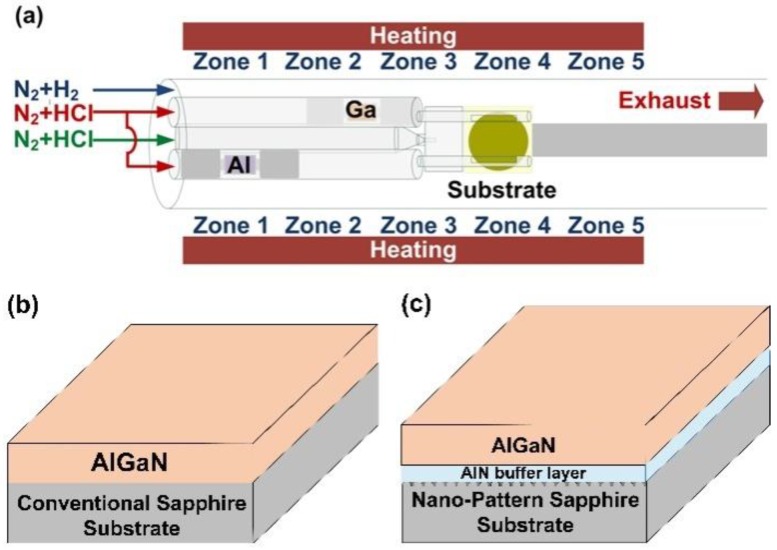
(**a**) A schematic diagram of the HVPE reactor used for the AlGaN grown on the (**b**) CSS and (**c**) AlN/NPSS templates.

A 2-inch c-plane sapphire substrate was used as a starting material for the NPSS. A SiO_2_ film deposited by low-pressure chemical vapor deposition on the sapphire served as the mask layer, on which the nanoimprint resist was then spin-coated. The hexagonal hole array was transferred to the resist by nanoimprint lithography, followed by oxygen plasma descum to remove any residual resistance at the bottom of the holes. The SiO_2_ film was then etched by fluorine plasma. Finally, a BCl_3_/Cl_2_ high-density plasma etching process was employed to etch the sapphire substrate, and the mask was removed by a dilute HF solution. Although multiple hole dimensions for nanoimprinting were attempted, the optimum NPSS used in this study was with 500 nm diameter hole arrays spaced 950 nm apart and etched to a depth of 400 nm. We deposited a 30 nm AlN buffer layer on the NPSS as an AlN/NPSS template using MOCVD, and then an AlGaN epilayer was grown on the AlN/NPSS template in an HVPE horizontal reactor as shown schematically in Figure 1a–c. For a 30 nm AlN thin film deposition, trimethylaluminum (TMAl, SAFC Hitech. Co., Ltd. Kaohsiung, Taiwan) and ammonia (NH_3_, SAFC Hitech. Co., Ltd. Kaohsiung, Taiwan) were used as the precursors. H_2_ was the carrier and the growth temperature at 1120 °C for 3 min. The AlGaN epilayer was also grown on a conventional sapphire substrate (CSS) as a comparison. The quartz glass reactor was covered with a furnace containing five heating zones maintained at different temperatures. Ga and Al metal chlorides serving as the group III Ga and Al precursor sources, respectively, were separately placed in the upstream region of the quartz reactor. The AlCl_3_ and GaCl vapors were generated in the reactor by flowing HCl (APDirect Inc. Co., Ltd. Taichung, Taiwan) over the Al (10 sccm) and Ga precursor (10 sccm) sources, respectively. To avoid the formation of AlCl vapor by a reaction between the Al metals and HCl at a high temperature (which would damage the quartz reactor), the Al metal source was maintained at 500 °C. The temperature of the GaCl source was maintained between 800 °C and 900 °C. Pure N_2_ gas (400 sccm) served as the carrier gas to propel the AlCl_3_ and GaCl vapors through the two quartz tubes to the growth zone. The ammonia line consisted of NH_3_ flow (2 L/min) and N_2_ flow (300 sccm). During the HVPE process, the H_2_ flow (Linde LienHwa Inc. Co., Ltd. Taipei, Taiwan) was kept at 2.45 L/min, N_2_ flow (Linde LienHwa Inc. Co., Ltd. Taipei, Taiwan) at 200 sccm, growth pressure at 200 mbar, and growth temperature at 1080 °C. 

Transmission electron microscopy (TEM; JEM-2010, JEOL, Tokyo, Japan), scanning electron microscopy (SEM; S-3000H, Hitachi, Tokyo, Japan), atomic force microscopy (AFM; 5400, Agilent, Santa Clara, CA, USA), double-crystal X-ray diffraction (DCXRD; X’Pert PRO MRD, PANalytical, Almelo, The Netherlands), and thin film stress (Toho, FLX-320-S, Nagoya, Japan) measurements were conducted to examine the microstructural properties of the AlGaN epilayers grown on the different substrate templates (e.g., CSS, AlN/NPSS, and NPSS).

## 3. Results and Discussion

**Figure 2:**
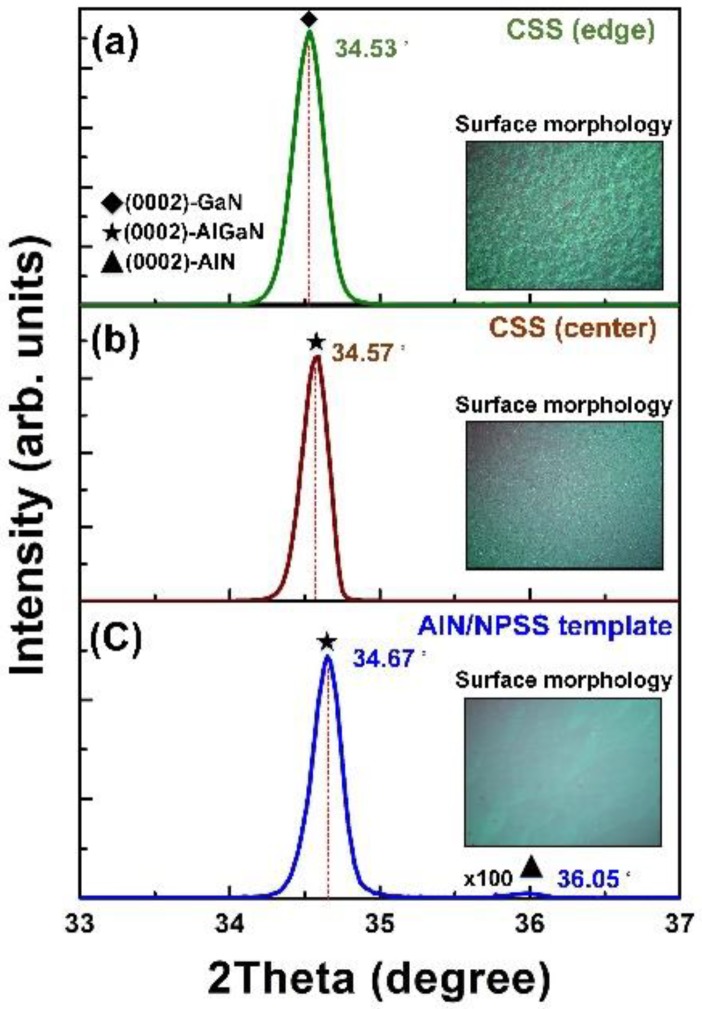
The typical XRD scan patterns of the AlGaN grown on (**a**) CSS (edge); (**b**) CSS (center); and (**c**) AlN/NPSS templates.

Figure 2 shows the typical XRD scan patterns of the AlGaN grown on the CSS and AlN/NPSS templates. To evaluate the influence of strain on the Al incorporation into the AlGaN layer, two different regions (the edge and the center of the two-inch wafer) in the AlGaN grown on the CSS wafer are also displayed for comparison. In Figure 2a, the peak located at 34.53° corresponds to the diffraction from the GaN (002) plane (i.e., edge of the wafer) on the CSS template. The AlGaN (002) peak located at 34.57° (very low Al content) was observed at the center of the wafer on the CSS template (Figure 2b). This was attributed to the residual strain that occurred due to the lattice mismatch between the AlGaN and the sapphire substrate. Meanwhile, in Figure 2c, the peak located at 34.67° corresponds to the AlGaN (002) plane, whereas a weak peak around 35.98° corresponds to the AlN (002) plane on the AlN/NPSS template. Apparently, the Al composition in the AlGaN epilayer on the CSS template was lower than that on the AlN/NPSS template (Al: 10%). This is because of the strain-dependent effect on the incorporation efficiency of Al into the AlGaN layer [27]. This result indicates that the improvement on the Al incorporation might be due to a change in the surface state caused by the introduction of the AlN/NPSS template during the growth of AlGaN. Moreover, the change in the composition of Al*_x_*Ga_1−*x*_N alloys might be due to the lattice mismatch or strain between the AlGaN and the sapphire’s rough film surface [28]. The insets in Figure 2a–c show the optical microscope morphologies of the AlGaN grown on CSS and AlN/NPSS templates, respectively. The AlGaN grown on the AlN/NPSS template exhibited the best morphology among the two other samples. It is believed that the introduction of the AlN/NPSS template was in favor of forming a smooth AlGaN film surface.

The crystal quality of these samples was also investigated using X-ray rocking curve (XRC) (plot is not shown). The XRC of the full-width at half-maximum (FWHM) value with the symmetric (002) and asymmetric (102) planes of the 3 μm thick AlGaN grown on the CSS and AlN/NPSS templates were evaluated, respectively. The FWHM values of the (002) and (102) planes of the AlGaN layer on the CSS template were estimated to be 2200 and 3600 arcsec, respectively. Meanwhile, the FWHM values of the (002) and (102) planes of the AlGaN grown on the AlN/NPSS template were 845 arcsec. These results indicate that the AlN/NPSS template improved the AlGaN layer’s crystal quality by lowering the dislocation density. It is well known that the symmetric (002) and asymmetric (102) reflections can provide some information on the density of pure screw and pure edge dislocations, respectively [29]. The relationship between the dislocation density and the FWHM values of XRC can be calculated using the following equations:

(1) $$\rho_{s}=\frac{\Delta \omega_{s}^{2}}{4.35c^{2}},\;\rho_{e}\frac{\Delta \omega_{e}^{2}}{4.35b^{2}},$$

where ρ_s_ and ρ_e_ are the screw and edge TD densities, respectively; the quantities of ω_s_ and ω_e_ refer to the FWHM of (002) and (102), respectively; *c* and *b* are the relevant Burgers vectors of the AlGaN epilayer. The corresponding dislocation densities of (002) and (102) reflections were determined using DCXRD as shown in Figure 2b. The AlGaN film on the AlN/NPSS template exhibited a lower screw dislocation density (1.4 × 10^9^ cm^−2^) than that on the CSS template (6.3 × 10^9^ cm^−2^). Therefore, it is believed that the AlN/NPSS template could reduce the residual tensile strain, leading to fewer defects, thus improving the quality of the AlGaN layer.

**Figure 3:**
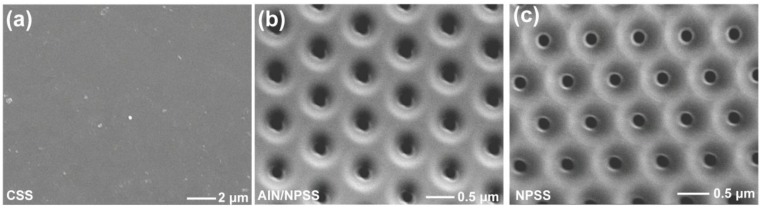
Top-view SEM images of the surface morphologies of the (**a**) CSS; (**b**) AlN/NPSS; and (**c**) NPSS [17].

**Figure 4:**
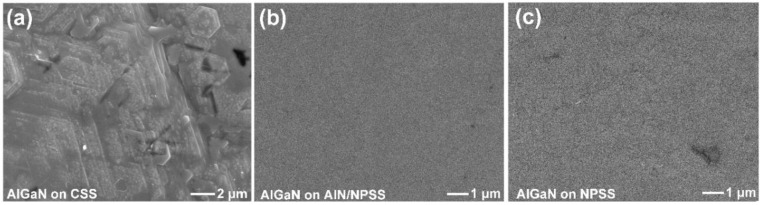
Top-view SEM images of the surface morphologies of the AlGaN epilayers grown on the (**a**) CSS; (**b**) AlN/NPSS; and (**c**) NPSS [17].

Figure 3a–c shows the top-view SEM images of CSS, AlN/NPSS, and NPSS [17], respectively. It can be seen that the prepared NPSS with hole patterns in this work, and the fabrication process is described in the method section. Figure 4a–c shows the top-view SEM images of the AlGaN layer grown on CSS, AlN/NPSS, and NPSS templates [17], respectively. Because of the lattice mismatch between the AlGaN and the CSS’s rough surface, incomplete 3D island coalescence with a hexagonal structure was formed (Figure 4a). On the other hand, the surface morphology of the AlGaN layer on the AlN/NPSS template was smooth and uniform (Figure 4b); the smooth surface might be due to the strain relaxation with a low defect density provided by the AlN/NPSS template. This observed result was consistent with that reported by Hagedorn et al. [18]. 

**Figure 5:**
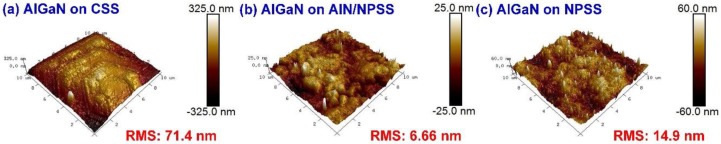
AFM measurements of the AlGaN grown on (**a**) CSS, (**b**) AlN/NPSS, and (**c**) NPSS [17] templates.

The corresponding surface roughness of these AlGaN samples was examined by AFM using a scan area of 10 μm^2^. As shown in Figure 5, the root mean square (RMS) values of the AlGaN/CSS, AlGaN/AlN/NPSS, and NPSS [17] were 79.1, 6.66, and 14.9, respectively. The large RMS value for the surface roughness of the AlGaN film grown on CSS (i.e., AlGaN/CSS) might be due to the large lattice mismatch between the film and the substrate. The decrease in the surface roughness was related to the reduction in the dislocation density, as mentioned in the DCXRD results. These observed results conclude that the structural properties and surface morphology of the AlGaN layer were mostly defined by the substrate template.

Since the lattice constant of the AlGaN epilayer is smaller than that of the sapphire, there exists tensile strain/stress of the AlGaN layer; thus, an AlN buffer layer is commonly used to compensate the tensile stress of the AlGaN grown on a sapphire substrate template [30]. To clearly understand the residual stress of the AlGaN layer, we estimated the strain (ε) present on the AlGaN epilayer from the FWHM of the major XRD (002) peak using the following equation [31]:

(2) $$\varepsilon =\frac{\beta }{4tan\theta }$$

where *β* is the FWHM and *θ* is Bragg’s diffraction angle. The calculated strain and stress are shown in Table 1. It should be noted that the stress of the AlGaN layer could be converted from tensile stress into compressive stress using the AlN/NPSS template.

**Figure 6:**
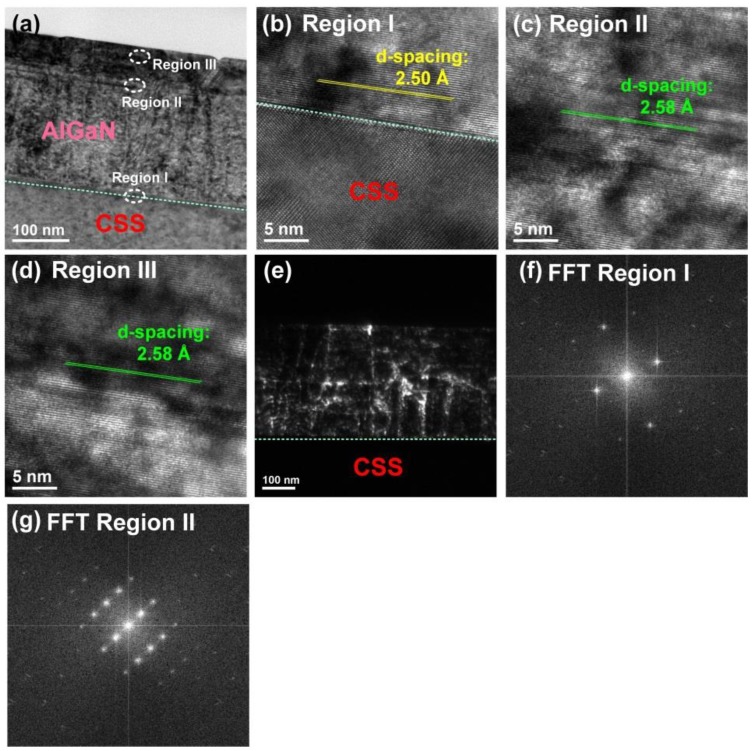
(**a**) A cross-sectional TEM image of the AlGaN/CSS sample. HRTEM images focused on (**b**) region I; (**c**) region II; and (**d**) region III as indicated in Figure 6a. (**e**) The dark-field TEM image observed in the two-beam condition for the AlGaN epilayer deposited on CSS. Fast Fourier transform images for regions (**f**) I and (**g**) II.

The TEM micrographs of the AlGaN deposited on the CSS template are shown in Figure 6. Figure 6a displays the cross-sectional TEM image of AlGaN on CSS, where the thickness of the AlGaN epilayer was is approximately 250 nm. To investigate the microstructures in more detail, we chose the three regions marked I, II, and III for high-resolution (HR) TEM measurements, as shown in Figure 6a,c,d, respectively. The HRTEM image of region I was taken at the interface between the AlGaN and the CSS. In this region, the *d*-spacing value of the epilayer was analyzed to be 2.50 Å. However, as shown in Figure 6c,d, a larger *d*-spacing value of 2.59 Å appeared in both regions II and III. According to the JCPDS database, the typical *d*-spacing values of GaN (0002) and AlN (0002) are 2.593 Å and 2.49 Å, respectively. The d-spacing is defined as the inter-atomic spacing or the distance between adjacent planes in the crystalline materials. From the analysis of region I (Figure 6a), the *d*-spacing value of 2.50 Å indicates that the AlGaN (0002) phase with a very high Al content was formed in the epilayer. Meanwhile, the *d*-spacing value of regions II and III (2.59 Å) was extremely close to that of the typical GaN (0002), revealing that the GaN (0002) phase also appeared in the epilayer. These TEM results were in good agreement with the XRD results (Figure 2a,b). This proof confirmed that the phase separation phenomenon between the GaN (0002) and the AlGaN (0002) phases indeed occurred in the AlGaN/CSS sample. This might be attributed to the in-plane stress caused by the phase separation of the AlGaN during growth. This observed result is also consistent with those reported by Gong et al. [32]. Additionally, the dark-field TEM image observed in the two beam condition for the AlGaN epilayer deposited on CSS is shown in Figure 6e, and the screw dislocation density of this AlGaN epilayer deduced by this TEM image is 7.7 × 10^9^ cm^−2^. Besides, the fast Fourier transform (FFT) images for regions I and II (shown in Figure 6a) are displayed in Figure 6f,g, respectively. The result can also prove that the phase separation exists in this AlGaN epilayer. 

**Figure 7:**
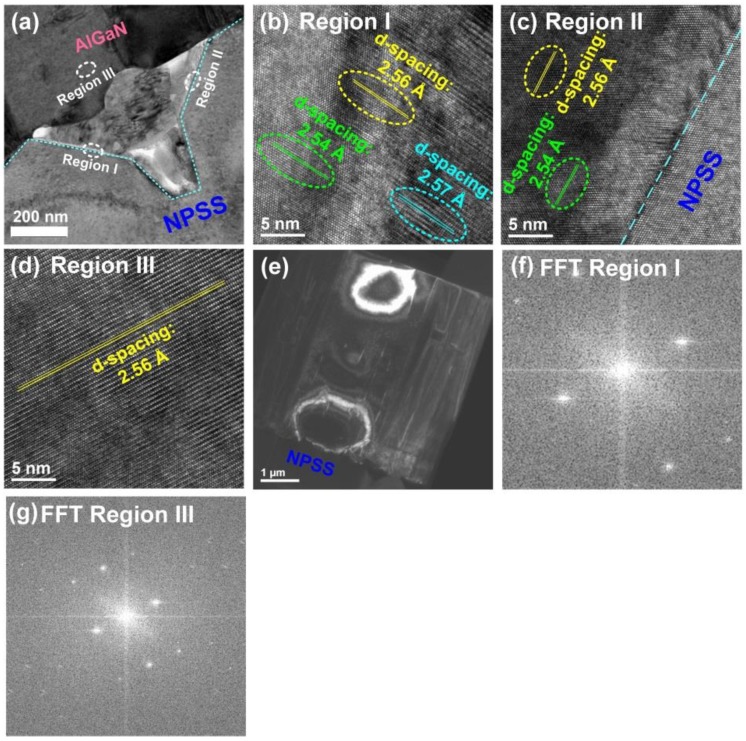
(**a**) A cross-sectional TEM image of the AlGaN/AlN/NPSS sample. HRTEM images focused on (**b**) region I; (**c**) region II; and (**d**) region III as indicated in Figure 7a. (**e**) The dark-field TEM image observed in the two beam condition for the AlGaN epilayer deposited on AlN/NPSS template. Fast Fourier transform images for regions (**f**) I and (**g**) III.

We also performed TEM measurements for the AlGaN epilayer deposited on the AlN/NPSS template, as shown in Figure 7. Figure 7a shows a cross-sectional TEM image of the AlGaN epilayer grown on the AlN/NPSS template, whereby the interface between the epilayer and the substrate was clearly observed. Although the AlN interfacial layer could not clearly been found in the present interface, it might be attributed to interdiffusion of Ga and Al during the growth process [33]. Three regions of the AlGaN epilayer (marked I, II, and III) were selected for the HRTEM measurements, as displayed in Figure 7b–d, respectively. Here, regions I and II both represented the AlGaN epilayers grown on the inclined planes (from different patterns). Meanwhile, region III represented the AlGaN epilayer grown above the top of the AlN/NPSS template. In Figure 7b, various *d*-spacing values consisting of 2.54 Å, 2.56 Å, and 2.57 Å were found in region I. Similar *d*-spacing values (2.54 Å and 2.56 Å) could also be identified in region II (Figure 7c). This reveals that the epilayer grown on the inclined planes (regions I and II) displayed the patterns belonging to the AlGaN (0002) phase. On the other hand, the *d*-spacing arrangement of the epilayer above the top of the AlN/NPSS template (region III) was more regular than that grown on the inclined planes, with one uniform *d*-spacing value of 2.56 Å. As mentioned above, the typical *d*-spacing value of GaN (0002) is 2.59 Å. Hence, the AlGaN epilayer deposited on the AlN/NPSS template indeed belonged to the AlGaN phase with no GaN phase, which agreed well with the XRD result. In addition, the dark-field TEM image observed in the two beam condition for the AlGaN epilayer deposited on AlN/NPSS template is shown in Figure 7e, and the screw dislocation density of this AlGaN epilayer deduced by this TEM image is 3.0 × 10^9^ cm^−2^. Based on Figure 6e and Figure 7e, it can be found that the screw dislocation densities of these two AlGaN epilayers deduced from these TEM images are indeed similar to those evaluated from the XRD results (Figure 2). Besides, the FFT images for regions I and III (shown in Figure 7a) are displayed in Figure 7f,g, respectively. The result can also prove that only the AlGaN phase (without GaN phase) is formed in this AlGaN epilayer. 

**Figure 8:**
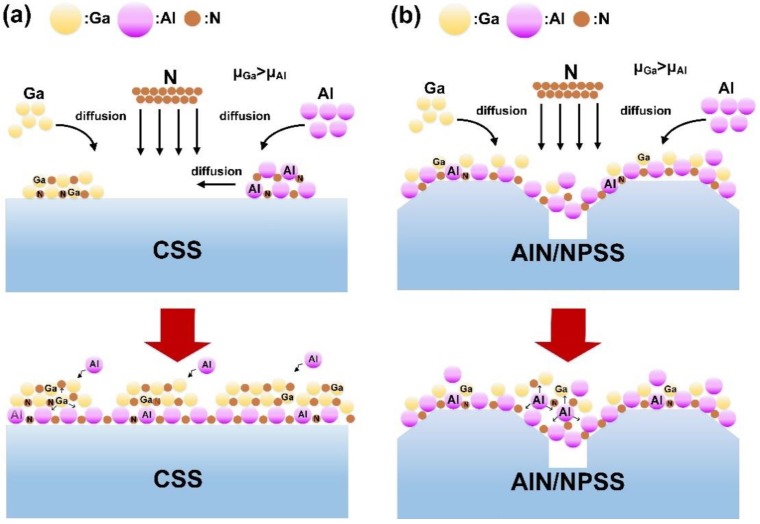
Schematic diagrams of the AlGaN growth mechanism on various substrates: (**a**) CSS and (**b**) AlN/NPSS.

Based on these observations, the mechanism of Al incorporation during the AlGaN growth was proposed, as schematically illustrated in Figure 8. In Figure 8a, due to the Ga atoms with high surface mobility, Ga atoms dominate the growth mechanisms and individual islands rapidly developed for GaN growth [34]. In Figure 8b, higher Al incorporation might be due to lower strain between the AlGaN film and the AlN/NPSS template [27]. It was also assumed that the slightly misorientated NPSS substrate could provide a better opportunity for the Al and Ga atoms to interact on the surface; hence, a higher Al composition of the AlGaN film was achieved. A similar result was also previously reported by Bryan et al. [35].

## 4. Conclusions

In this study, the effects of different substrate templates on the structural and stress properties of AlGaN epilayers growth by HVPE were investigated. According to the XRD, AFM, and TEM analyses, the Al incorporation efficiency into the AlGaN epilayer could be increased using the AlN/NPSS template. The surface roughness of the layer could also be suppressed by growing the AlGaN layer on the AlN/NPSS template. As a result, we could obtain a relatively high Al content and smooth AlGaN film with a narrow XRD FWHM and low defect density. These results indicated that HVPE AlGaN/AlN/NPSS could be a promising epitaxial template for the development of high-performance AlGaN-based optoelectronics devices.

## Figures and Tables

**Table 1:** Strain (ε) and stress (σ) of the AlGaN layer grown on CSS or AlN/NPSS.

AlGaN-(002)	Substrate	2 Theta (°)	FWHM (°)	ε	σ (MPa)
	CSS	34.57	0.583	−1.6 × 10^−5^	1187
	AlN/NPSS	34.65	0.235	−4.7 × 10^−5^	38.41
